# Supplementary material for: COZOID: contact zone identifier for visual analysis of protein-protein interactions
Source: BMC Bioinformatics. 2018 Apr 6;19:125. doi: 10.1186/s12859-018-2113-6 (PMC5889581; doi:10.1186/s12859-018-2113-6)
Supplement: Supplementary file 2 — Software build. Executable binary file of the software tool. (ZIP 118,784 kb) [file 12859_2018_2113_MOESM2_ESM.zip › cozoid/java/docs/beansbinding-1.2.1-doc.zip!/org/jdesktop/el/CompositeELResolver.html]

CompositeELResolver (Beans Binding)


---


|  |  |  |
| --- | --- | --- |
| **Overview** \| **Package** \| **Class** \| **Use** \| **Tree** \| **Deprecated** \| **Index** \| **Help** | |  |
| **PREV CLASS**   **NEXT CLASS** | **FRAMES**    **NO FRAMES**     **All Classes** |  |
| SUMMARY: NESTED \| FIELD \| CONSTR \| METHOD | DETAIL: FIELD \| CONSTR \| METHOD |  |


---


## org.jdesktop.el Class CompositeELResolver

```
java.lang.Object
  org.jdesktop.el.ELResolver
      org.jdesktop.el.CompositeELResolver
```

---

``` public class CompositeELResolver extends ELResolver ```

Maintains an ordered composite list of child `ELResolver`s.

Though only a single `ELResolver` is associated with an
`ELContext`, there are usually multiple resolvers considered
for any given variable or property resolution. `ELResolver`s
are combined together using a `CompositeELResolver`, to define
rich semantics for evaluating an expression.

For the `getValue(org.jdesktop.el.ELContext, java.lang.Object, java.lang.Object)`, `getType(org.jdesktop.el.ELContext, java.lang.Object, java.lang.Object)`, `setValue(org.jdesktop.el.ELContext, java.lang.Object, java.lang.Object, java.lang.Object)` and
`isReadOnly(org.jdesktop.el.ELContext, java.lang.Object, java.lang.Object)` methods, an `ELResolver` is not
responsible for resolving all possible (base, property) pairs. In fact,
most resolvers will only handle a `base` of a single type.
To indicate that a resolver has successfully resolved a particular
(base, property) pair, it must set the `propertyResolved`
property of the `ELContext` to `true`. If it could
not handle the given pair, it must leave this property alone. The caller
must ignore the return value of the method if `propertyResolved`
is `false`.

The `CompositeELResolver` initializes the
`ELContext.propertyResolved` flag to `false`, and uses
it as a stop condition for iterating through its component resolvers.

The `ELContext.propertyResolved` flag is not used for the
design-time methods `getFeatureDescriptors(org.jdesktop.el.ELContext, java.lang.Object)` and
`getCommonPropertyType(org.jdesktop.el.ELContext, java.lang.Object)`. Instead, results are collected and
combined from all child `ELResolver`s for these methods.

**Since:**
:   JSP 2.1

**See Also:**: `ELContext`, `ELResolver`

---

**Field Summary**

**Fields inherited from class org.jdesktop.el.ELResolver**

`RESOLVABLE_AT_DESIGN_TIME, TYPE`


**Constructor Summary**

`CompositeELResolver()`


| **Method Summary** | |
| --- | --- |
| `void` | `add(ELResolver elResolver)`             Adds the given resolver to the list of component resolvers. |
| `java.lang.Class<?>` | `getCommonPropertyType(ELContext context, java.lang.Object base)`             Returns the most general type that this resolver accepts for the `property` argument, given a `base` object. |
| `java.util.Iterator<java.beans.FeatureDescriptor>` | `getFeatureDescriptors(ELContext context, java.lang.Object base)`             Returns information about the set of variables or properties that can be resolved for the given `base` object. |
| `java.lang.Class<?>` | `getType(ELContext context, java.lang.Object base, java.lang.Object property)`             For a given `base` and `property`, attempts to identify the most general type that is acceptable for an object to be passed as the `value` parameter in a future call to the `setValue(org.jdesktop.el.ELContext, java.lang.Object, java.lang.Object, java.lang.Object)` method. |
| `java.lang.Object` | `getValue(ELContext context, java.lang.Object base, java.lang.Object property)`             Attempts to resolve the given `property` object on the given `base` object by querying all component resolvers. |
| `boolean` | `isReadOnly(ELContext context, java.lang.Object base, java.lang.Object property)`             For a given `base` and `property`, attempts to determine whether a call to `setValue(org.jdesktop.el.ELContext, java.lang.Object, java.lang.Object, java.lang.Object)` will always fail. |
| `void` | `setValue(ELContext context, java.lang.Object base, java.lang.Object property, java.lang.Object val)`             Attempts to set the value of the given `property` object on the given `base` object. |

**Methods inherited from class java.lang.Object**

`clone, equals, finalize, getClass, hashCode, notify, notifyAll, toString, wait, wait, wait`

**Constructor Detail**

### CompositeELResolver

```
public CompositeELResolver()
```


**Method Detail**

### add

```
public void add(ELResolver elResolver)
```

:   Adds the given resolver to the list of component resolvers.

    Resolvers are consulted in the order in which they are added.

    :   **Parameters:**: `elResolver` - The component resolver to add. **Throws:**: `java.lang.NullPointerException` - If the provided resolver is `null`.

---


### getValue

```
public java.lang.Object getValue(ELContext context,
                                 java.lang.Object base,
                                 java.lang.Object property)
```

:   Attempts to resolve the given `property` object on the given
    `base` object by querying all component resolvers.

    If this resolver handles the given (base, property) pair,
    the `propertyResolved` property of the
    `ELContext` object must be set to `true`
    by the resolver, before returning. If this property is not
    `true` after this method is called, the caller should ignore
    the return value.

    First, `propertyResolved` is set to `false` on
    the provided `ELContext`.

    Next, for each component resolver in this composite:

    1. The `getValue()` method is called, passing in
       the provided `context`, `base` and
       `property`.
    2. If the `ELContext`'s `propertyResolved`
       flag is `false` then iteration continues.
    3. Otherwise, iteration stops and no more component resolvers are
       considered. The value returned by `getValue()` is
       returned by this method.

    If none of the component resolvers were able to perform this
    operation, the value `null` is returned and the
    `propertyResolved` flag remains set to
    `false`

    .

    Any exception thrown by component resolvers during the iteration
    is propagated to the caller of this method.

    :   **Specified by:**: `getValue` in class `ELResolver`
    :   **Parameters:**: `context` - The context of this evaluation.: `base` - The base object whose property value is to be returned, or `null` to resolve a top-level variable.: `property` - The property or variable to be resolved. **Returns:**: If the `propertyResolved` property of `ELContext` was set to `true`, then the result of the variable or property resolution; otherwise undefined. **Throws:**: `java.lang.NullPointerException` - if context is `null`: `PropertyNotFoundException` - if the given (base, property) pair is handled by this `ELResolver` but the specified variable or property does not exist or is not readable.: `ELException` - if an exception was thrown while performing the property or variable resolution. The thrown exception must be included as the cause property of this exception, if available.

---


### getType

```
public java.lang.Class<?> getType(ELContext context,
                                  java.lang.Object base,
                                  java.lang.Object property)
```

:   For a given `base` and `property`, attempts to
    identify the most general type that is acceptable for an object to be
    passed as the `value` parameter in a future call
    to the `setValue(org.jdesktop.el.ELContext, java.lang.Object, java.lang.Object, java.lang.Object)` method. The result is obtained by
    querying all component resolvers.

    If this resolver handles the given (base, property) pair,
    the `propertyResolved` property of the
    `ELContext` object must be set to `true`
    by the resolver, before returning. If this property is not
    `true` after this method is called, the caller should ignore
    the return value.

    First, `propertyResolved` is set to `false` on
    the provided `ELContext`.

    Next, for each component resolver in this composite:

    1. The `getType()` method is called, passing in
       the provided `context`, `base` and
       `property`.
    2. If the `ELContext`'s `propertyResolved`
       flag is `false` then iteration continues.
    3. Otherwise, iteration stops and no more component resolvers are
       considered. The value returned by `getType()` is
       returned by this method.

    If none of the component resolvers were able to perform this
    operation, the value `null` is returned and the
    `propertyResolved` flag remains set to
    `false`

    .

    Any exception thrown by component resolvers during the iteration
    is propagated to the caller of this method.

    :   **Specified by:**: `getType` in class `ELResolver`
    :   **Parameters:**: `context` - The context of this evaluation.: `base` - The base object whose property value is to be analyzed, or `null` to analyze a top-level variable.: `property` - The property or variable to return the acceptable type for. **Returns:**: If the `propertyResolved` property of `ELContext` was set to `true`, then the most general acceptable type; otherwise undefined. **Throws:**: `java.lang.NullPointerException` - if context is `null`: `PropertyNotFoundException` - if the given (base, property) pair is handled by this `ELResolver` but the specified variable or property does not exist or is not readable.: `ELException` - if an exception was thrown while performing the property or variable resolution. The thrown exception must be included as the cause property of this exception, if available.

---


### setValue

```
public void setValue(ELContext context,
                     java.lang.Object base,
                     java.lang.Object property,
                     java.lang.Object val)
```

:   Attempts to set the value of the given `property`
    object on the given `base` object. All component
    resolvers are asked to attempt to set the value.

    If this resolver handles the given (base, property) pair,
    the `propertyResolved` property of the
    `ELContext` object must be set to `true`
    by the resolver, before returning. If this property is not
    `true` after this method is called, the caller can
    safely assume no value has been set.

    First, `propertyResolved` is set to `false` on
    the provided `ELContext`.

    Next, for each component resolver in this composite:

    1. The `setValue()` method is called, passing in
       the provided `context`, `base`,
       `property` and `value`.
    2. If the `ELContext`'s `propertyResolved`
       flag is `false` then iteration continues.
    3. Otherwise, iteration stops and no more component resolvers are
       considered.

    If none of the component resolvers were able to perform this
    operation, the `propertyResolved` flag remains set to
    `false`

    .

    Any exception thrown by component resolvers during the iteration
    is propagated to the caller of this method.

    :   **Specified by:**: `setValue` in class `ELResolver`
    :   **Parameters:**: `context` - The context of this evaluation.: `base` - The base object whose property value is to be set, or `null` to set a top-level variable.: `property` - The property or variable to be set.: `val` - The value to set the property or variable to. **Throws:**: `java.lang.NullPointerException` - if context is `null`: `PropertyNotFoundException` - if the given (base, property) pair is handled by this `ELResolver` but the specified variable or property does not exist.: `PropertyNotWritableException` - if the given (base, property) pair is handled by this `ELResolver` but the specified variable or property is not writable.: `ELException` - if an exception was thrown while attempting to set the property or variable. The thrown exception must be included as the cause property of this exception, if available.

---


### isReadOnly

```
public boolean isReadOnly(ELContext context,
                          java.lang.Object base,
                          java.lang.Object property)
```

:   For a given `base` and `property`, attempts to
    determine whether a call to `setValue(org.jdesktop.el.ELContext, java.lang.Object, java.lang.Object, java.lang.Object)` will always fail. The
    result is obtained by querying all component resolvers.

    If this resolver handles the given (base, property) pair,
    the `propertyResolved` property of the
    `ELContext` object must be set to `true`
    by the resolver, before returning. If this property is not
    `true` after this method is called, the caller should ignore
    the return value.

    First, `propertyResolved` is set to `false` on
    the provided `ELContext`.

    Next, for each component resolver in this composite:

    1. The `isReadOnly()` method is called, passing in
       the provided `context`, `base` and
       `property`.
    2. If the `ELContext`'s `propertyResolved`
       flag is `false` then iteration continues.
    3. Otherwise, iteration stops and no more component resolvers are
       considered. The value returned by `isReadOnly()` is
       returned by this method.

    If none of the component resolvers were able to perform this
    operation, the value `false` is returned and the
    `propertyResolved` flag remains set to
    `false`

    .

    Any exception thrown by component resolvers during the iteration
    is propagated to the caller of this method.

    :   **Specified by:**: `isReadOnly` in class `ELResolver`
    :   **Parameters:**: `context` - The context of this evaluation.: `base` - The base object whose property value is to be analyzed, or `null` to analyze a top-level variable.: `property` - The property or variable to return the read-only status for. **Returns:**: If the `propertyResolved` property of `ELContext` was set to `true`, then `true` if the property is read-only or `false` if not; otherwise undefined. **Throws:**: `java.lang.NullPointerException` - if context is `null`: `PropertyNotFoundException` - if the given (base, property) pair is handled by this `ELResolver` but the specified variable or property does not exist.: `ELException` - if an exception was thrown while performing the property or variable resolution. The thrown exception must be included as the cause property of this exception, if available.

---


### getFeatureDescriptors

```
public java.util.Iterator<java.beans.FeatureDescriptor> getFeatureDescriptors(ELContext context,
                                                                              java.lang.Object base)
```

:   Returns information about the set of variables or properties that
    can be resolved for the given `base` object. One use for
    this method is to assist tools in auto-completion. The results are
    collected from all component resolvers.

    The `propertyResolved` property of the
    `ELContext` is not relevant to this method.
    The results of all `ELResolver`s are concatenated.

    The `Iterator` returned is an iterator over the
    collection of `FeatureDescriptor` objects returned by
    the iterators returned by each component resolver's
    `getFeatureDescriptors` method. If `null` is
    returned by a resolver, it is skipped.

    :   **Specified by:**: `getFeatureDescriptors` in class `ELResolver`
    :   **Parameters:**: `context` - The context of this evaluation.: `base` - The base object whose set of valid properties is to be enumerated, or `null` to enumerate the set of top-level variables that this resolver can evaluate. **Returns:**: An `Iterator` containing zero or more (possibly infinitely more) `FeatureDescriptor` objects, or `null` if this resolver does not handle the given `base` object or that the results are too complex to represent with this method **See Also:**: `FeatureDescriptor`

---


### getCommonPropertyType

```
public java.lang.Class<?> getCommonPropertyType(ELContext context,
                                                java.lang.Object base)
```

:   Returns the most general type that this resolver accepts for the
    `property` argument, given a `base` object.
    One use for this method is to assist tools in auto-completion. The
    result is obtained by querying all component resolvers.

    The `Class` returned is the most specific class that is
    a common superclass of all the classes returned by each component
    resolver's `getCommonPropertyType` method. If
    `null` is returned by a resolver, it is skipped.

    :   **Specified by:**: `getCommonPropertyType` in class `ELResolver`
    :   **Parameters:**: `context` - The context of this evaluation.: `base` - The base object to return the most general property type for, or `null` to enumerate the set of top-level variables that this resolver can evaluate. **Returns:**: `null` if this `ELResolver` does not know how to handle the given `base` object; otherwise `Object.class` if any type of `property` is accepted; otherwise the most general `property` type accepted for the given `base`.


---


|  |  |  |
| --- | --- | --- |
| **Overview** \| **Package** \| **Class** \| **Use** \| **Tree** \| **Deprecated** \| **Index** \| **Help** | |  |
| **PREV CLASS**   **NEXT CLASS** | **FRAMES**    **NO FRAMES**     **All Classes** |  |
| SUMMARY: NESTED \| FIELD \| CONSTR \| METHOD | DETAIL: FIELD \| CONSTR \| METHOD |  |


---
